# Developing and validating a scale to measure Food and Nutrition Literacy (FNLIT) in elementary school children in Iran

**DOI:** 10.1371/journal.pone.0179196

**Published:** 2017-06-27

**Authors:** Aazam Doustmohammadian, Nasrin Omidvar, Nastaran Keshavarz-Mohammadi, Morteza Abdollahi, Maryam Amini, Hassan Eini-Zinab

**Affiliations:** 1 Department of Community Nutrition, National Nutrition and Food Technology Research Institute; and Faculty of Nutrition Sciences and Food Technology, Shahid Beheshti University of Medical Sciences, Tehran, Iran; 2 School of Public Health, Shahid Beheshti University of Medical Sciences, Tehran, Iran; 3 Department of Nutrition Research, National Nutrition and Food Technology Research Institute; and Faculty of Nutrition Sciences and Food Technology, Shahid Beheshti University of Medical Sciences, Tehran, Iran; IRCCS Istituto Auxologico Italiano, ITALY

## Abstract

**Background:**

Food and nutrition literacy is an emerging term which is increasingly used in policy and research. Though research in this area is growing, progression is limited by the lack of an accepted method to measure food and nutrition literacy. The aim of this study is to develop a valid and reliable questionnaire to assess food and nutrition literacy in elementary school children in the city of Tehran.

**Methods:**

The study was conducted in three phases. To develop Food and Nutrition Literacy (FNLIT) questionnaire, a comprehensive literature review and a qualitative study were initially performed to identify food and nutrition literacy dimensions and its components. Content and face validity of the questionnaire were evaluated by an expert panel as well as students. In the second phase, construct validity of the scale was evaluated using Explanatory Factor Analyses (EFA) and Confirmatory Factor Analyses (CFA). In the last phase (confirmatory phase), the final version of the questionnaire was evaluated on 400 students.

**Results:**

Findings show Content Validity Ratio (CVR) and Content Validity Index (CVI) of the 62-item questionnaire at acceptable levels of 0.87 and 0.92, respectively. EFA suggested a six-factor construct, namely, understanding food and nutrition information, knowledge, functional, interactive, food choice, and critical. The results of CFA indicated acceptable fit indices for the proposed models. All subscales demonstrated satisfactory internal consistency (Cronbach’s alpha≥0.70), except for critical skill subscale (0.48). The intraclass correlation coefficient (ICC = 0.90, CI: 0.83–0.94) indicated that Food and Nutrition Literacy (FNLIT) scale had satisfactory stability. Each phase of development progressively improved the questionnaire, which resulted in a 46-item (42 likert-type items and 4 true-false items) Food and Nutrition Literacy (FNLIT) scale. The questionnaire measured two domains with 6 subscales, including: 1) cognitive domain: understanding and knowledge; 2) skill domain: functional, food choice, interactive, and critical skills.

**Conclusion:**

The developed food and nutrition literacy scale is a valid and reliable instrument to measure food and nutrition literacy in children. This measure lays a solid empirical and theoretical foundation for future research and tailored interventions to promote food and nutrition literacy in this age group.

## Introduction

Non-Communicable Diseases (NCDs), including obesity, diabetes, cardiovascular diseases (CVDs) and hypertension are the leading causes of premature death worldwide. Of these premature deaths, 80% occur in low and middle income countries [1]. According to the World Health Organization (WHO), smoking and sedentary behaviors along with unhealthy dietary intake are common risk factors for 80% of chronic diseases [2]. Furthermore, the focus on the role of nutrition in the etiology of chronic diseases is increasing [2]. Nutrition transition has resulted in a great change in dietary habits of children and adolescents throughout the world [3,4]. Due to time constraints, families have started to rely on convenience and pre-packed foods, which are usually high in saturated fats, sugar and salt with limited consumption of fruits, vegetables and fiber [5,6,7]. In Iran, as a country experiencing nutrition transition, high-risk behaviors such as unhealthy dietary intake [8,9] and physical inactivity [10,11] are rising, resulting in an increase in the prevalence of childhood overweight and obesity [11,12].

According to studies, children’s food choices and dietary habits can affect the risk of nutrition-related diseases lifelong [13,14]. Childhood, therefore provides an opportunity that can be utilized by health promoters to establish healthy behaviors that could prevent the development of health problems later in life [15,16]. Understanding determinants of unhealthy behaviors is therefore crucial. One way for understanding the reasons behind the nutrition-related problems and behaviors among children and adolescents is assessment of their food and nutrition literacy level [17].

Food literacy is an emerging term defined as “collection of inter-related knowledge, skills and behaviors required to plan, manage, select, prepare and eat foods to meet needs and determine food intake”[18]. This term is increasingly used in nutrition related policy and research to address complex health problems. Therefore, improving children’s food and nutrition literacy has been in particular the target of intervention studies and contemporary nutrition plans and policies [19]. Though research in this area is growing, progression is limited by the lack of an accepted method to measure food and nutrition literacy. Development of a scale to assess children’s food and nutrition literacy level therefore, is required to guide the development and ensure effectiveness of nutrition related interventions [19]. The present study aimed to develop and validate a questionnaire to assess food and nutrition literacy in 10–12 years old children in Tehran.

## Materials and methods

### Theoretical framework

This study was based on Nutbeam’s hierarchical model for health literacy [20]. Nutbeam proposed two distinctly different conceptual approaches for health literacy: health literacy as a “risk factor” and health literacy as an “asset”. The first approach needs to be identified and appropriately managed in clinical care. The second approach has evolved from origins in public health and health promotion [20]. Using such insights, health literacy can be categorized into different levels that progressively reflect greater autonomy and personal empowerment in decision making, as well as engagement in a wider range of health actions that extend from personal behaviors to social action to address the determinants of health [21]. According to Nutbeam’s hierarchical model, nutrition literacy is the ability to access, interpret and use nutrition information [22]. Nutrition literacy can be classified in three levels as functional, interactive and critical [23]. At the lowest level, functional nutrition literacy is concerned with basic reading and writing skills necessary to understand and follow simple nutrition message(s). The second level, interactive nutrition literacy, is advanced literacy which includes cognitive and interpersonal skills needed to manage nutrition issues in partnership with professionals. As an example of second level actions one can refer to ability of students to interact nutritional information with others (peer, family and nutritionists) in order to promote healthy eating pattern. Finally, the third level, critical nutrition literacy, is the ability to analyze nutrition information critically, increase awareness, and participate in action to address barriers. Examples of this level are engagement of students to oppose opening of a fast food restaurant near their school; and community participation in order to promote healthy eating pattern [23,24]. The second and third levels are in a hierarchical order.

### Study design

**Fig 1:**
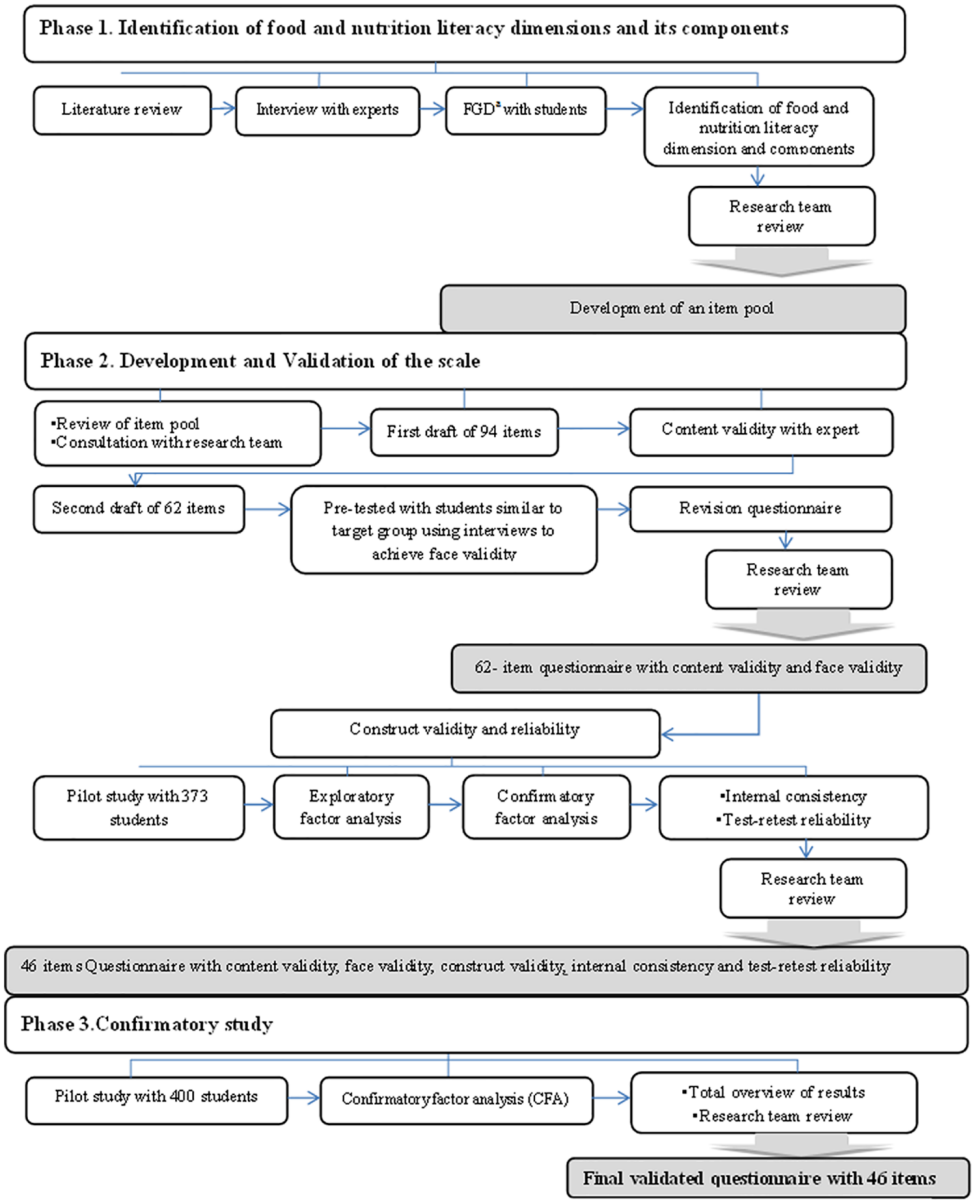
Summary of steps followed in the development of the food and nutrition literacy scale. ^a^ Focus Group Discussion.

The study was designed in three distinct phases, aimed at ensuring validity and reliability: 1) identification of food and nutrition literacy dimensions and its components; 2) development and validation of a scale; and 3) confirmatory study to ensure validity of the scale. Fig 1, presents an overview of scale development process.

#### Phase1: Identification of food and nutrition literacy dimensions and its components (scale items)

1. **Literature review.** A comprehensive literature search through PubMed, ISI, Science direct, Scopus and Google Scholar was conducted to identify concepts of food and nutrition literacy and its components, as well as related questionnaires using the Keywords: ”food skill”, “food literacy”, “nutrition literacy”, “health literacy”, “food preparation”, “food choice“, “food wellbeing”, for the first search. In the second search, reference lists of the studies were checked for additional related works.
2. **Qualitative study**. Using a qualitative approach, 15 in-depth interviews with experts and 12 focus group discussions with 10–12 years old students (n = 89, mean age = 11.07 years) were conducted to explore their perceptions about food and nutrition literacy concepts. Data were open coded by two authors independently to look for key themes and components of food and nutrition literacy. Transcripts were reviewed at least 5 times. All coding and interpretations were discussed by the research team. Interviewing stopped when theoretical saturation reached. Data were analyzed using MAXQDA_2010_ software.

#### Phase2: Development and validation of the scale

1. **Item generation.** Using the concepts identified at phase one and reviewing existing questionnaires [25,26,27,28,29,30], a pool of 103 items was generated to measure 5 domains and 12 components of food and nutrition literacy. After elimination of redundant items, 94 items remained which included 90 Likert-type and 4 true/false items. To assess construct validity, factor analyses was performed only on likert-type items.
2. **Content validity.** For qualitative content validity, a panel of eight experts (3 nutritionists, 2 health education and health promotion, 2 sociologists, 1 social medicine and 1 public health professionals) examined the initial questionnaire. Items were modified based on the experts’ comments. To calculate content validity ratio (CVR) and content validity index (CVI), the experts were asked to comment on the necessity, relevance, clarity and simplicity of each item. A CVR for total scale was computed according to Lawshe scores [31]. The CVI of each question was determined by the proportion of experts who rated each item with a 3 or a 4 [32]. Content validity and expert panel review led to elimination of 32 items. The second draft of the scale consisted of 62 items, including4 true-false and 58 likert-type items.
3. **Face validity.** To confirm face validity of the scale, 15 students aged 10–12 years, similar to target group, were recruited through convenience sampling. Students were interviewed to assess each item for ambiguity and complexity.
4. **Construct validity.** For construct validity, 373 students aged 10–12 years, participated in the study during October 2015. The General Office of Education in Tehran classifies existing 19-educational-districts into three socioeconomic levels, including: affluent (Northern Tehran), semi-affluent (Central Tehran) and deprived (Southern Tehran). To maximize heterogeneity of the sample, two schools were randomly selected from each of the three different socio-economic levels (a total of 6 schools). A second round of random sampling was used to select students from the schools. Written informed consent was obtained from students. Data analysis for construct validity included the following two phases:
  - **Exploratory factor analysis (EFA).** To assess construct validity of the scale, EFA was used to explore whether the statements in the scale reflected the three levels of nutrition literacy based on Nutbeam’s hierarchical model of health literacy. An oblique rotation (i.e. promax) and Principal Axis Factoring (PAF) extraction were used to explore the existing factorial pattern. The number of factors was determined through evaluating four criteria: eigenvalues, percent of explained variance by each factor, scree plot and interpretability criteria [33]. Based on this evaluation, a decision on the number of factors was made. The decision to delete items was based on the item’s factor loading.
  - **Confirmatory factor analysis (CFA).** Confirmatory factor analysis was performed to test whether data fit the hypothesized measurement model, which was extracted by EFA.
5. **Reliability**. Reliability of the scale was assessed using internal consistency reliability and test-retest procedure. Internal consistency of subscales was evaluated by calculating Cronbach’s alpha for each scale. For reproducibility, test-retest was performed by re-administration of the questionnaire on 30 students aged 10–12 years, (15 girls and 15 boys), two weeks apart. Average length of time for completion of the questionnaire was 20 minutes. At the end of this phase, the final draft of the questionnaire with 46 items (42 likert-type and 4 true-false) was developed.

#### Phase 3: Confirmatory study

In order to evaluate the factor structures identified through this analysis, 400 students aged 10–12 years, were selected from three different socio-economic areas: districts 2, 4 and 5 (affluent areas); districts 9, 11 and 14 (semi-affluent areas) and districts 15, 16 and 17 (deprived areas) of Tehran Metropolitan Area. To assess consistency of results, the selected samples were different from those studied in the construct validity study. Written informed consent was obtained from students and their parents. Data collection conducted during November 2015 to January 2016. Confirmatory factor analysis was performed by AMOS using the same parameters and fit indices as phase 2.

### Statistical analysis

Exploratory Factor Analysis (EFA) was used to determine the number and nature of underlying factors in the scale. Kaiser-Meyer-Olkin (KMO) was used to measure sampling adequacy. Bartlett’s test of sphericity, and total variance explained were used for the evaluation of factor analysis. An oblique rotation (i.e. promax) and Principal Axis Factoring (PAF) extraction were used in the EFA. Factor loadings were used to keep or drop items. Confirmatory Factor Analysis (CFA) was performed to test whether the data fit the hypothesized measurement model, which was extracted by EFA. Weighted Least Squares (WLS) estimation method was used at CFA. Asymptomatic covariance matrix was considered as a weighted matrix. Goodness-of-fit indices (GFIs) and reasonable threshold levels of these indices for CFA were considered as χ2/df< 3, root mean square error of approximation (RMSEA) < 0.08, goodness-of-fit index (GFI)> 0.9 and adjusted goodness of fit index (AGFI)>0/8 [34]. Internal consistency of likert-type items of the scale was determined by calculating Cronbach’s alpha coefficient. Kuder-Richardson formula 20 (KR-20) was used for true-false items. Values equal to 0.7 and above were considered as satisfactory [35]. Before the Cronbach’s alpha calculation, coding for reverse- items were reversed. The test-retest reliability of the scale was evaluated using the intraclass correlation coefficient (ICC) where ICCs > 0.75 were considered acceptable. The test-retest reliability of true-false items was evaluated by Cohen kappa coefficient. Kappa values greater than 0.75 were defined as excellent accord, and those below 0.5 as poor [36]. All statistical analysis were performed using SPSS 21.0 (SPSS Inc., Chicago, Illinois, U.S.) and AMOS 21.0 [37].

### Ethics statement

The study protocol was approved by the National Nutrition and Food Technology Research Institute (NNFTRI) ethical committee (No.1394.20, 16-10-2015). Informed written consent was obtained from children and their parents.

## Results

### Phase 1: Dimensions of food and nutrition literacy and its components

1. **Literature review.** A total of thirty studies were included in the review. Of these, 5 studies simultaneously addressed both food/nutrition literacy definitions and its components [18,22,25,38,39], only 17 studies defined food/nutrition literacy [19,40,41,42,43,44,45,46,47,48,49,50,51,52,53,54,55] and 8 focused on food/nutrition literacy dimensions [17,24,56,57,58,59,60,61]. Based on the literature, components of nutrition literacy are based on Nutbeam’s concept of health literacy. They are mainly focused on abilities necessary to obtain, understand and process food and nutrition information. The components of food literacy incorporated a broader spectrum of skills. Most of food literacy literature emphasized on abilities and skills in three domains as **food knowledge** and understanding the effects of food on health; **skills** needed to make healthy food choices and preparation, and **capacities**, including self-efficacy and creativity.
2. **Qualitative study**. Nutbeam’s hierarchical model of health literacy was the theoretical framework used to develop skill domain of measurement. In order to assess theoretical framework in our local context, a qualitative study was conducted with food and nutrition experts, as well as students. Based on the results of qualitative study, in cognitive domain, 2 dimensions, including *knowledge* and *understanding* were identified. In skill domain in line with Nutbeam’s hierarchical model of health literacy, 3 dimensions, including *functional*, *interactive* and *critical* literacy were identified. In general, 12 components of food and nutrition literacy identified which fell into five dimensions. Table 1 presents these results.

**Table 1:** Food and nutrition literacy dimensions and components in children.

Domain	Dimensions	Components
**Cognitive**	Knowledge	Food and nutrition knowledge
		Lifestyle knowledge
		Food safety knowledge
	Understanding	Understanding food and nutrition information
**Skills**	Functional	Access
		Applying
		a. Healthy eating behaviors and health
		b. Food choices
	Interactive	Interactive skills
		Emotional skills
		Discussion Skills
	Critical	Media literacy
		Analysis of food labeling
		Decision-making and planning

### Phase 2: Development and validation of the scale

**Fig 2:**
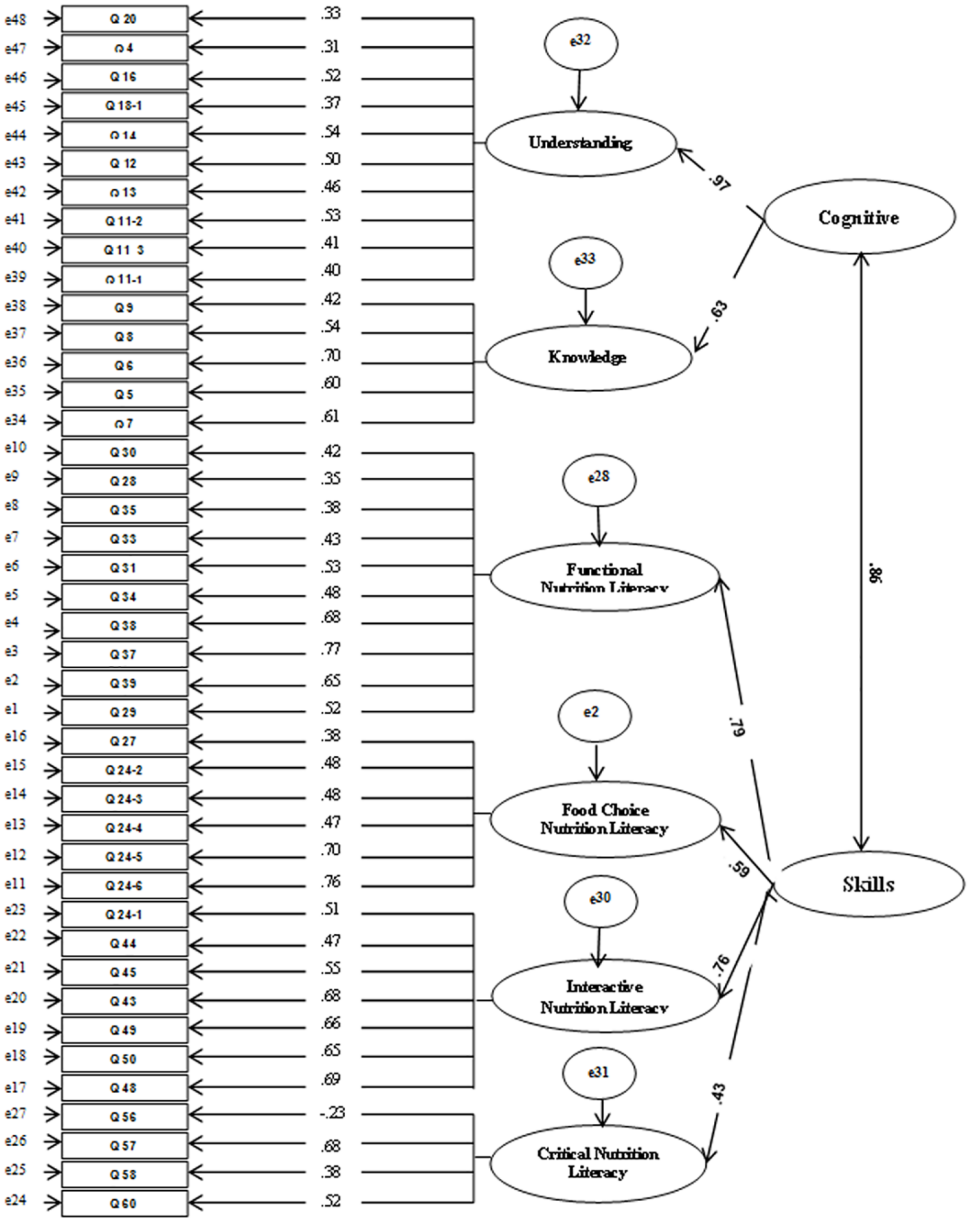
Second-order confirmatory factor analysis factor loadings construct validity study for FNLIT scale. Note: n = 373; All factor loadings are standardized and are statistically significant, p< 0.001.

1. **Item generation.** A pool of 103 items was generated at first phase of the study. After elimination of redundant items, 94 items remained. They included 90 Likert-type items to assess the five dimensions of food and nutrition literacy and 4 true/false items to assess food label literacy.
2. **Content validity.** Findings regarding the CVR and CVI confirmed the quantitative content validity of 62 items. The CVR for total scale was 0.87, indicating a satisfactory result [62]. A satisfactory level of agreement was found (CVI = 0.92) among panelists suggesting that the scale had a good content validity [63].
3. **Face validity.** Based on the results of face validity, most of the scale items were generally easy to read and comprehend for students; except for a few words that were changed to meet participants’ considerations, such as replacing “**rarely**” with “**seldom**” as recommended by the children.
  Through content validity, experts commented on the necessity, relevance, clarity and simplicity of each item. Following the experts’ assessments, face validity was achieved through interview with students similar to target group to assess each items for ambiguity and complexity. Receiving an acceptable level of content and face validity in the second phase ensured that questionnaire items are tailored for the study group. In addition, through two further studies with 773 students (373 students in construct validity study and 400 students in confirmatory study), the questionnaire was completed under researchers supervision. During these studies the respondents did not encountere any complex or ambiguous item, which also justified the results of validity study.
4. **Construct validity**. A total of 373 students participated in the construct validity study, %51 of which were male. The average age of students was 11.07±0.57 years. Participants were from grades 5 (48.3%) and 6 (51.7%). Demographic characteristics of students participated in the second phase (construct validity study) are shown at Table 2.
  - **Exploratory factor analysis (EFA).** For cognitive domain, the Kaiser–Meyer–Olkin (KMO) test showed sampling adequacy (KMO = 0.78), and Bartlett’s test confirmed factor analysis was appropriate (χ2 = 1241.35, df = 231, and P < 0.001). Two factors (understanding and knowledge) with 17 items were extracted for cognitive domain. In skills domain of food and nutrition literacy, KMO showed sampling adequacy (KMO = 0.85), and Bartlett’s test confirmed the EFA was appropriate (χ2 = 3385.36, df = 630, and P < 0.001). This domain, consistent with the theoretical hypotheses, included four factors with29 items (i.e. functional, interactive, food choice and critical). Factor loading, eigenvalue, explained variance percent and croanbach’s α related to cognitive and skill domains are reported in S1 File. Additional Alpha test be deleting items one at a time showed that removing items Q18_1, Q18_3, Q40 and Q36 of understanding, knowledge, functional and interactive subscales resulted in an increase in Cronbach’s alpha (S1 and S2 Tables). Considering items content and their factor loadings, items Q18_3 and Q36 were removed which resulted in a significant increase in the corresponding sub-scale's Cronbach’s alpha from 0.63 to 0.69 and 0.70 to 0.79, respectively. Also, these items poorly represented the core constructs and their elimination was justifiable to the research team. After removing the specified items, scales were re-analyzed. The KMO sampling adequacies were greater than 0.80 (KMO = 0.81 in cognitive domain and KMO = 0.84 in skill domain) and the Bartlett Sphericity Test was significant at p< 0. 001. The final EFA extracted two factors with 15 items in cognitive domain (Table 3). The percentage of the total variance was 23.72% by the two rotated factors. In skills domain, four factors, including 27 items were extracted (Table 4). The percentage of total variance explained by these factors was 32.97%. The final results of EFA and the internal consistency of items are presented in Tables 3 and 4. As shown, alpha for the subscales would not be improved by removing any item. All items were loaded between 0.22 and 0.64 for cognitive domain and between 0.30 and 0.75 for skills domain.
  - **Confirmatory factor analysis (CFA).** The result of CFA showed the first-order factor loadings for cognitive domain of scale ranged from 0.29 to 0.70, and for skills domain of scale ranged from 0.23 to 0.78 (S1 and S2 Figs). All factor loadings were statistically significant (p<0.001). Fig 2, displays the standardized factor loadings for the second-order factor model in construct validity study. The results of the model fit for the first-order factor models of cognitive and skills domains of scale are reported in Table 5 which indicates desirable fit of the proposed models.
5. **Reliability**. Internal consistency reliabilities and Cronbach’s alpha for each of the subscales are presented in Table 6. Cronbach’s alpha coefficient ranged from 0.48 to 0.80 for various domains. Kuder-Richarson reliability index for the dichotomous responses of food label critical literacy was acceptable (0.71). Kappa coefficients for each pair of dichotomous responses of items 43, 44, 45 and 46 were at acceptable levels of 0.68, 0.92, 0.83, and 0.85, respectively. The intraclass correlation coefficient (ICC = 0.90) indicated that Food and Nutrition Literacy (FNLIT) scale had satisfactory stability.

**Table 2:** Demographic characteristics of students participated in validity and confirmatory studies.

**construct validity study (n = 373)**						
**Characteristics**	**Girls (n = 181)**			**Boys (n = 192)**		
	**Grade 5**^**th**^	**Grade 6**^**th**^	**Total**	**Grade 5**^**th**^	**Grade 6**^**th**^	**Total**
	**(n = 85)**	**(n = 96)**		**(n = 105)**	**(n = 97)**	
	**N (%)**	**N (%)**	**N (%)**	**N (%)**	**N (%)**	**N (%)**
**Districts in the city**						
**North (district 2)**	27 (31.8)	30 (31.2)	57 (31.5)	33 (31.4)	34 (35)	67 (34.9)
**Center (district 9)**	33 (38.8)	30 (31.2)	63 (34.8)	36 (34.3)	29 (29.9)	65 (33.8)
**South (district 19)**	25 (29.4)	36 (37.5)	61(33.7)	26 (24.7)	34 (35)	60 (31.2)
**confirmatory study (n = 400)**						
**Characteristics**	**Girls (n = 196)**			**Boys(n = 204)**		
	**Grade 5**^**th**^	**Grade 6**^**th**^	**Total**	**Grade 5**^**th**^	**Grade 6**^**th**^	**Total**
	**(n = 99)**	**(n = 97)**		**(n = 100)**	**(n = 104)**	
	**N (%)**	**N (%)**	**N (%)**	**N (%)**	**N (%)**	**N (%)**
**Districts in the city**						
**North (districts 2. 4, 5)**	39 (39.4)	39 (40.2)	78 (39.8)	34 (34)	41 (39.4)	75 (36.8)
**Center (districts 9,11,14)**	37 (37.4)	32 (33)	69 (35.2)	38 (38)	35 (33.7)	73 (35.8)
**South (districts 15,16,17)**	23 (33.2)	26 (26.8)	49(25)	28 (28)	28 (26.9)	56 (27.5)

**Table 3:** Factor analysis results and item statistics of cognitive domain of food and nutrition literacy after item deleted because of increasing alpha in students aged 10–12 (n = 373).

		EFA factor loadings of cognitive domain		
scale items, subscales, and total		Understanding	Knowledge	α if item deleted
**1. Understanding**				
Q11_1	When shopping, how important is the nutritional information about food ingredients for you?	**0.586**	-0.058	0.676
Q11_3	When shopping, how important is standardized labeling on food packages for you?	**0.577**	-0.205	0.691
Q11_2	When shopping, how important are production and expiration dates for you?	**0.564**	0.071	0.679
Q13	I can easily understand the nutrition facts (e.g. amount of energy, sugar, protein, etc.) on food packages.	**0.467**	-0.038	0.680
Q12	I can easily understand nutritional issues I read about in newspapers, magazines, and brochures.	**0.449**	0.021	0.675
Q14	I can understand nutritionists’ recommendations about health and nutritional requirements that are appropriate for my age group.	**0.432**	0.120	0.674
Q18_1	Boiling is one of the more healthy cooking methods.	**0.386**	0.010	0.692
Q16	I can understand information and recommendations about proper nutrition for children in the media (e.g., TV, Internet, radio, etc.)	**0.356**	0.192	0.677
Q4	Daily physical activity for 30–40 minutes prevents obesity.	**0.274**	0.110	0.701
Q20	I know how different vegetables are cultivated and grown.	**0.226**	0.058	0.708
Q3	Daily eating breakfast helps me to learn more.	0.213	0.188	-
Q10	Unhealthy food packing without standard sign and health license not to be used.	0.208	0.183	-
**2. Knowledge**				
Q7	Consumption of salty snacks (e.g. chips, corn puffs, etc.) is harmful for health.	-0.050	**0.638**	0.614
Q5	Excessive consumption of sugar, sweets, and chocolate is harmful for health.	-0.115	**0.623**	0.635
Q6	Consumption of salami and sausage that are high in fat may cause obesity.	0.096	**0.613**	0.599
Q8	Consumption of salamis and sausages may cause cancer.	-0.027	**0.562**	0.642
Q9	Reading of production and expiration date on food package is important for health.	0.192	**0.289**	0.689
**Eigenvalue**		**3.85**	**1.59**	**-**
**Explained Variance (%)**		**18.40**	**5.31**	**-**
**Cronbach’s α total**		**0.71**	**0.69**	**-**

**Table 4:** Factor analysis results and item statistics of skills domains of food and nutrition literacy after item deleted because of increasing alpha in students (N = 373).

		EFA factor loadings of skills domain				
scale items, subscales, and total		Functional nutrition literacy	Interactive nutrition literacy	Food choice nutrition literacy	Critical nutrition literacy	α if item deleted
**1. Functional**						
Q29	I eat a variety of vegetables (e.g., lettuce, cabbage, tomatoes, carrots, etc.), every day.	**0.655**	-0.219	0.094	0.051	0.772
Q39	I share the nutritional issues that I obtain from various sources with others (e.g., friends, family, etc.)	**0.615**	0.050	-0.033	-0.053	0.765
Q37	I talk to my friends and family about healthy eating.	**0.601**	0.240	0.043	-0.121	0.754
Q38	If I have any questions about food and nutrition issues, I’m able to get information and advice from parents, teachers, etc.	**0.542**	0.122	-0.016	0.068	0.766
Q34	I prepare my own snacks for school.	**0.524**	0.088	-0.081	-0.120	0.774
Q31	I bring healthy snacks to school.	**0.522**	0.019	0.026	-0.069	0.774
Q33	I regularly do exercise or walk for 30 to 40 minutes every day.	**0.479**	-0.032	0.008	0.036	0.782
Q35	I wash and prepare fruits and vegetables myself.	**0.475**	0.047	-0.148	-0.128	0.786
Q28	I eat fruits every day.	**0.467**	-0.173	-0.054	0.315	0.786
Q30	I eat breakfast every day	**0.446**	-0.032	0.043	-0.023	0.784
**2. Interactive**						
Q48	I have enough power to resist unhealthy foods (e.g., fast food, pizza, carbonated drinks, etc.)	-0.041	**0.746**	-0.017	-0.013	0.755
Q50	If I go to restaurant or fast food with my friends, and all of them choose unhealthy foods (e.g. pizza, French fries, carbonated drinks, etc.), I’m able to choose healthy foods.	-0.044	**0.708**	-0.020	-0.044	0.762
Q49	I can easily say “no” to any unhealthy eating suggestions from my friends.	0.052	**0.629**	-0.014	0.027	0.757
Q43	If I encounter unhealthy behaviors at home, school, or in other settings, I’m able to challenge them.	0.083	**0.576**	0.021	0.093	0.761
Q45	If my parents or family prepare unhealthy snacks (e.g., chips, fruit roll-ups, corn snacks, etc.) for me to take to school, I accept them.	0.049	**0.537**	-0.143	0.117	0.792
Q44	If may family were overweight and eating a high fat diet, I would tell them to change their eating habits.	-0.093	**0.511**	0.126	-0.100	0.778
Q24_1	When I go shopping with my mother or father, I buy healthy snacks such as nuts, raisins, and dried chickpeas instead of chips, snacks, chocolate, and sweets.	0.030	**0.346**	0.271	0.125	0.789
Q67	I manage my schedules in the way to be able to do exercise for half an hour every day.	0.231	0.231	0.035	0.129	-
**3. Food choice**						
Q24_6	When I go shopping with my mother or father, I buy foods that are certified as healthy.	0.003	0.066	**0.750**	-0.149	0.664
Q24_5	When I go shopping with my mother or father, I buy foods with standardized labeling.	0.171	-0.022	**0.637**	-0.161	0.683
Q24_4	When I go shopping with my mother or father, I buy foods that are not expired.	-0.190	-0.050	**0.613**	0.096	0.689
Q24_3	When I go shopping with my mother or father, I buy foods with sustainable packaging.	-0.081	0.030	**0.539**	0.023	0.691
Q24_2	When I go shopping with my mother or father, I will buy foods that are stored appropriately or kept refrigerated.	0.050	-0.013	**0.440**	0.138	0.689
Q27	I eat food from all the food groups every day.	0.126	-0.094	**.306**	0.241	0.718
**4. Critical**						
Q60	I usually try new foods that I’ve never eaten.	-0.094	-0.101	0.023	**0.556**	0.489
Q57	I usually try new vegetables that I’ve never eaten.	0.003	0.075	0.094	**0.431**	0.320
Q58	I can buy healthy food from the school cafeteria, depending on my pocket money.	0.013	0.322	0.014	**0.343**	0.428
Q56	If school cafeteria doesn’t offer any healthy foods, it will be difficult for me to choose a healthy snack.	0.102	-0.120	0.081	**-0.306**	0.401
**Eigenvalue**		**6.23**	**2.42**	**1.76**	**1.45**	**-**
**Explained Variance (%)**		**20.00**	**6.35**	**3.96**	**2.66**	**-**
**Cronbach’s α total**		**0.80**	**0.80**	**0.73**	**0.48**	**-**

**Table 5:** Results of confirmatory factor analysis for the first-order and second-order model of FNLIT scale ^a^.

Model		Χ2	df	P value	Χ2/df	GFI	AGFI	RMSEA
**First-order model**	Cognitive domain	192.05	89	0.000	2.15	0.92	0.90	0.05
	skills domain	768.53	320	0.000	2.40	0.87	0.84	0.06
**Second-order model**	construct validity study	1566.94	814	0.000	1.92	0.83	0.81	0.05
	confirmatory study	2120.75	817	0.000	2.59	0.81	0.78	0.06

^a^Abbreviations: FNLIT = Food and Nutrition Literacy; χ2, Chi square; df, degree of freedom; GFI, goodness fit index; AGFI, adjusted goodness of fit index; RMSEA, root mean square error of approximation.

**Table 6:** Croanbach’s α coefficient and ICC for the FNLIT scale and its subscales.

		Number of items	Croanbach’s α	ICC^a^ (95%CI)
			N = 373	N = 30
**Cognitive domain**	1. Understanding	10	0.71	0.84(0.73–0.91)
	2. Knowledge	5	0.69	0.80 (0.68–0.89)
**Skills domain**	1. Functional	10	0.80	0.87 (0.79–0.93)
	2. Interactive	7	0.80	0.91 (0.87–0.95)
	3. Food choice	6	0.73	0.80 (0.68–0.89)
	4. Critical	4	0.48	0.78 (0.63–0.78)
**Food and Nutrition Literacy Scale (total)**		42		0.89 (0.83–0.94)

^**a**^ICC = intraclass correlation coefficient; FNLIT = Food and Nutrition Literacy.

### Phase 3: Confirmatory study

400 students (51% male) aged 10–12 years (11.3±0.65) participated in the confirmatory study. Demographic characteristics of students participated in confirmatory study are demonstrated in Table 2. The higher-order factor model was run in new sample and demonstrated desirable fit as indicated by multiple fit indicators shown in Table 5. Factor loadings for the second-order factor model in confirmatory study ranged from 0.40 to 0.90 and all factor loadings were statistically significant (p < 0.001).

## Discussion

Through this study a reliable and valid instrument to assess food and nutrition literacy in elementary school students was developed. To our knowledge, this study is the first attempt to develop and evaluate a food and nutrition literacy scale in children using a mixed method. Several studies have developed nutrition knowledge questionnaires in children, but not nutrition literacy [64,65,66,67,68]. It’s rarely been the case where children’s food and nutrition literacy explored through both expert and children’s perspectives [25,30]. Measuring food and nutrition literacy is a new issue. Existing food and nutrition literacy instruments tend to emphasize literacy and numeracy skills, as well as nutrition knowledge [26,27] mainly in adults. Some researchers have just applied nutrition knowledge-based outcomes in their work [58,69], whereas others have focused on the ability to effectively use food labels [27,30]. Studies suggest that food and nutrition literacy is a social, cultural and political feature that should be considered as a structure with multiple dimensions [18,24]. The complex nature of food and nutrition literacy concept confirms the necessity to use a multi-dimensional tool. The designed questionnaire included a wide range of items to assess individual, interpersonal, and social factors relating to children’s food and nutrition literacy. The questionnaire led to a comprehensive approach of functional, interactive and critical dimension in a diverse context.

Since the skill domain of scale was based on theoretical framework, we independently analyzed the variables of cognitive and skills domains. During exploratory factor analysis, it was tried to remain loyal to the conceptual framework of the study. Although we originally proposed three factors of the skill domain of food and nutrition literacy scale, explanatory factor analysis supported a four-factor model. The factors or subscales of skill domain which conceived by the research team represented functional, food choice, interactive and critical skills of food and nutrition literacy scale. This may suggest that food choice is the most important part of children’s food and nutrition literacy which have potential to influence their food consumption decisions [18,70]. In estimating dimensions of model, due to lack of consensus among Structural Equation Model (SEM) specialists, several model fit indices were used [71], including chi-square (χ^2^), goodness-of-fit index (GFI), the adjusted goodness-of-fit index (AGFI) and root mean square error of approximation (RMSEA). Cut-points of model fit criteria show acceptable values for the first-order models of cognitive and skill domains of food and nutrition literacy scale. Although, GFI was very close to the nominal value of 0.9 [71] for skill domain. Therefore, two-factor structure of cognitive domain and four-factor structure of skill domain of food and nutrition literacy scale was confirmed. In the second-order model, the GFI of both construct validity and confirmatory analysis were close to the nominal value of 0.9. This result can be interpreted as a GFI that may have been affected by the external factors such as, sample size, the number of parameters and the degrees of freedom to sample size ratio and does not reflect poor model fit [72,73]. In our study, the degree of freedom was more than sample size which warranted this result. The RMSEA is currently the most popular measure of fitness which was within the acceptable range both at first-order and second-order models [71]. Therefore, results of structural equation models showed the optimum model of the scale was matched to the theoretical approach and reasonably confirmed by related indices.

All of the subscales demonstrated satisfactory test-retest reliability and their internal consistency reliabilities generally exceeded the standard of 0.70, except for critical skill subscale. This finding was consistent with result of Ndahura (2012), which also found the internal consistency value of Critical Nutrition Literacy (CNL) construct (0.46) lower than the standard value [25]. A possible explanation for the low internal consistency values is that internal consistency reliabilities values depend on the number of items in the scale. Since the critical skill subscale consisted of four items, this could have resulted in lower internal consistency values [74]. The lower reliability observed for this subscale may reflect the variability observed in the scores of the items comprising it. The lower reliability estimates will not necessarily negate the value of the subscale, since expert panel rated the items as relevant during the second phase. After the completion of validity and reliability phases, the food and nutrition literacy scale for children consisted of 4 true-false items and 42 likert-type items within 6 areas. These were understanding and knowledge, as well as functional, food choice, interactive and critical food and nutrition literacy.

The strength of the current study is that food and nutrition literacy scale was developed and validated based on a theoretical framework, using mixed method approach. Furthermore, the final food and nutrition scale was confirmed through re-analysis in a new independent data set which empirically supported the reasonable fit of the model. The results also provide insight regarding the dimensions of food and nutrition literacy. However, the study has certain limitations. First, a predictability criterion to report was not used. In addition, considering the fact that food and nutrition literacy is influenced by culture and society, it is important to consider the role of deeply rooted sociocultural norms regarding health and eating. Therefore, the questionnaire cannot serve as a universal tool, and should be localized according to social context [24]. The study focused on a specific age group and school settings, future studies are encouraged to be carried out among different age groups of children and in different settings. The evaluation of such studies may lead to a stronger confirmation of the validity properties of the FNLIT scale. At present, both Persian and English versions of the questionnaire are available (S1 and S2 Files).

## Conclusion

Food and nutrition literacy scale is a valid and reliable instrument to measure food and nutrition literacy in children in Iran. This measure lays a solid empirical and theoretical foundation for future research and tailored interventions to promote food and nutrition literacy in children.

## Supporting information

S1 Fig First-order confirmatory factor analysis factor loadings construct validity study for cognitive domain of FNLIT scale. Note: n = 373; All factor loadings are standardized and are statistically significant, p< 0.001.
(TIF)

S2 Fig First-order confirmatory factor analysis factor loadings construct validity study for skill domain of FNLIT scale. Note: n = 373; All factor loadings are standardized and are statistically significant, p< 0.001.
(TIF)

S1 Table Factor analysis results and item statistics of cognitive domain of food and nutrition literacy in students aged 10–12 (N = 373). ^a^ Item deleted because of increasing alpha.
(DOCX)

S2 Table Factor analysis results and item statistics of cognitive and skills domains of food and nutrition literacy in students (N = 373). ^a^ Item deleted because of increasing alpha.
(DOCX)

S1 File Food and Nutrition Literacy (FNLIT) scale_English version. (PDF)

S2 File Food and Nutrition Literacy (FNLIT) scale_Persian version. (PDF)

S3 File Data. (SAV)
