# Supplementary figures and images for: Developing and validating a scale to measure Food and Nutrition Literacy (FNLIT) in elementary school children in Iran
Source: PLoS One. 2017 Jun 27;12(6):e0179196. doi: 10.1371/journal.pone.0179196 (PMC5487019; doi:10.1371/journal.pone.0179196)

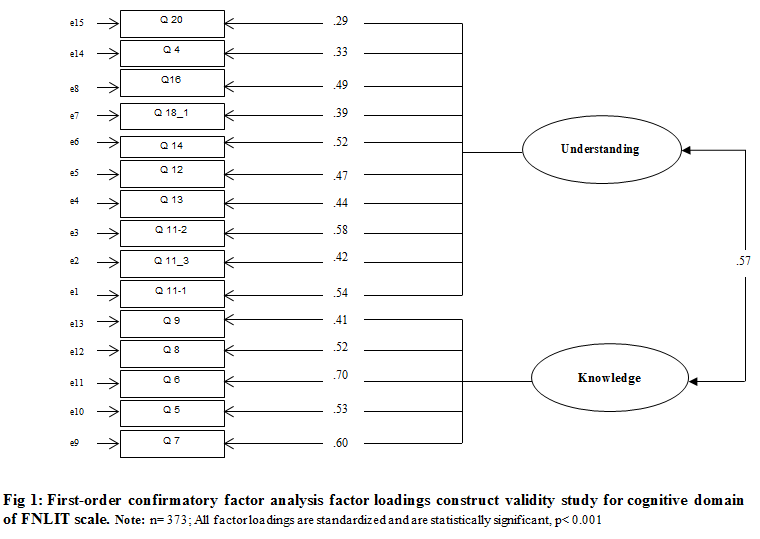

Supplement: S1 Fig — Note: n = 373; All factor loadings are standardized and are statistically significant, p< 0.001. (TIF) [file pone.0179196.s001.tif]

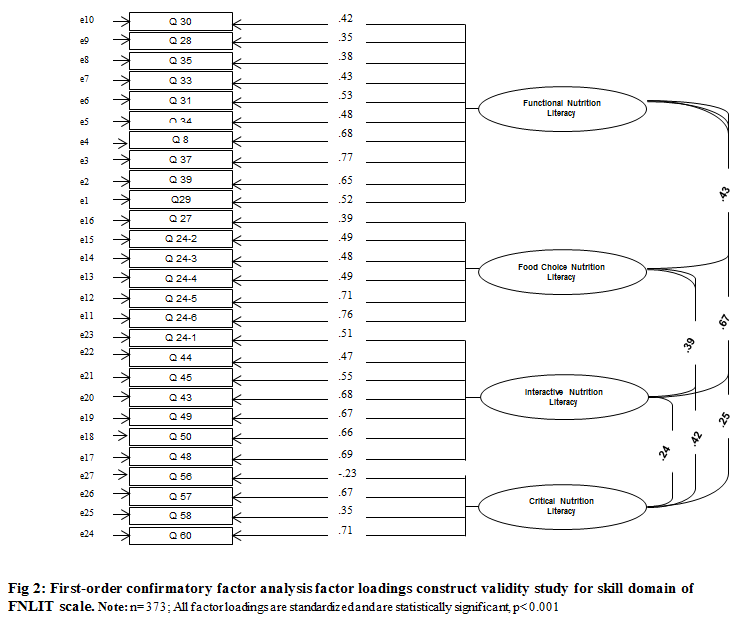

Supplement: S2 Fig — Note: n = 373; All factor loadings are standardized and are statistically significant, p< 0.001. (TIF) [file pone.0179196.s002.tif]
