# Supplementary material for: Developing and validating a scale to measure Food and Nutrition Literacy (FNLIT) in elementary school children in Iran
Source: PLoS One. 2017 Jun 27;12(6):e0179196. doi: 10.1371/journal.pone.0179196 (PMC5487019; doi:10.1371/journal.pone.0179196)
Supplement: S1 Table — a Item deleted because of increasing alpha. (DOCX) [file pone.0179196.s003.docx]

|  | | **EFA factor loadings of cognitive domain** | |  |
| --- | --- | --- | --- | --- |
| **scale items, subscales, and total** | | **Understanding** | **Knowledge** | **α**  **if item deleted** |
| 1. **Understanding** | |  |  |  |
| Q11_1 | When shopping, how important is the nutritional information about food ingredients for you? | **0.578** | -0.056 | 0.674 |
| Q13 | I can easily understand the nutrition facts (e.g. amount of energy, sugar, protein, etc.) on food packages. | **0.529** | -0.102 | 0.674 |
| Q11_2 | When shopping, how important are the production and expiration dates for you? | **0.516** | 0.099 | 0.676 |
| Q11_3 | When shopping, how important is standardized labeling on food packages for you? | **0.492** | -0.134 | 0.687 |
| Q14 | I can understand nutritionists’ recommendations about health and nutritional requirements that are appropriate for my age group. | **0.475** | 0.064 | 0.668 |
| Q12 | I can easily understand nutritional issues I read in newspapers, magazines and brochures. | **0.445** | 0.029 | 0.675 |
| Q18_1 | Boiling is one of the more healthy cooking methods. | **0.412** | -0.011 | 0.686 |
| Q16 | I can understand information and recommendations about proper nutrition for children in the media (e.g. TV, internet, radio, etc.,) | **0.388** | 0.156 | 0.674 |
| Q18_2 | Sautee is one of the more healthy cooking methods | **0.321** | -0.262 | 0.713^a^ |
| Q4 | Daily physical activity for 30- 40 minutes prevents obesity. | **0.264** | 0.121 | 0.692 |
| Q20 | I know how different vegetables are cultivated and grown. | **0.252** | 0.036 | 0.703 |
| Q10 | Unhealthy food packed without standardized labeling and health license not to be used. | **0.244** | 0.150 | 0.694 |
| Q17 | When I read food and nutrition issues in internet, magazine, newspaper or book, I faced with words that are difficult for me. | 0.135 | -0.111 | - |
| Q2 | No food exclusively can meet all my nutritional needs. | 0.121 | 0.047 | - |
| **2. knowledge** | |  |  |  |
| Q7 | Consumption of salty snacks (e.g., chips, corn puffs, etc.) is harmful for health. | -0.059 | **0.671** | 0.547 |
| Q5 | Excessive consumption of sugar, sweets and chocolate is harmful for health. | -0.080 | **0.594** | 0.577 |
| Q6 | Consumption of salami and sausage that are high in fat may cause obesity. | 0.147 | **0.553** | 0.555 |
| Q8 | Consumption of salami and sausage may cause cancer. | -0.005 | **0.544** | 0.588 |
| Q18_3 | Frying is one of the more healthy cooking methods. | -0.132 | **0.309** | 0.683^a^ |
| Q9 | Reading of production and expiration dated on food packaged is important for health. | 0.197 | **0.286** | 0.622 |
| Q3 | Daily eating breakfast helps me to learn more. | 0.187 | **0.223** | 0.628 |
| Q15 | I need other’s help to understand nutritional issues in internet, magazine and book,. | -0.092 | -0.175 | **-** |
| **Eigenvalue** | | **3.96** | **1.75** | **-** |
| **Explained Variance (%)** | | **14.65** | **4.54** | **-** |
| **Croanbach’s α** | | **0.70** | **0.63** | **-** |

^a^ Item deleted because of increasing alpha

**S1 Table. Factor analysis results and item statistics of cognitive domain of food and nutrition literacy in students aged 10-12 (n=373)**

^a^ Item deleted because of increasing alpha
