# Supplementary material for: Developing and validating a scale to measure Food and Nutrition Literacy (FNLIT) in elementary school children in Iran
Source: PLoS One. 2017 Jun 27;12(6):e0179196. doi: 10.1371/journal.pone.0179196 (PMC5487019; doi:10.1371/journal.pone.0179196)
Supplement: S2 Table — a Item deleted because of increasing alpha. (DOCX) [file pone.0179196.s004.docx]

| ^a^ Item deleted because of increasing alpha  **S2 Table. Factor analysis results and item statistics of skills domains of food and nutrition literacy in students (N=373)** | | | **EFA factor loadings of skills domain** | | | |  |
| --- | --- | --- | --- | --- | --- | --- | --- |
| **scale items, subscales, and total** | | | **Functional nutrition literacy** | **Interactive nutrition literacy** | **Food choice nutrition literacy** | **Critical nutrition literacy** | **α**  **if item deleted** |
| 1. **Functional nutrition literacy** | | |  |  |  |  |  |
| Q39 | I share the nutritional issues that I obtain from various sources with others (e.g., friends, family, etc.) | | **0.678** | -0.005 | -0.008 | -0.091 | 0.775 |
| Q37 | I talk to my friends and family about healthy eating. | | **0.671** | 0.195 | 0.029 | -0.112 | 0.765 |
| Q29 | I eat a variety of vegetables (e.g., lettuce, cabbage, tomatoes, carrots, etc.), every day. | | **0.648** | -0.249 | 0.144 | 0.010 | 0.781 |
| Q34 | I prepare my own snacks for school. | | **0.570** | 0.049 | -0.096 | -0.108 | 0.783 |
| Q38 | If I have any questions about food and nutrition issues, I’m able to get information and advice from parents, teachers, etc. | | **0.557** | 0.069 | 0.015 | 0.071 | 0.774 |
| Q35 | I wash and prepare fruits and vegetables myself. | | **0.549** | 0.000 | -0.152 | -0.158 | 0.793 |
| Q31 | I bring healthy snacks to school. | | **0.503** | 0.039 | 0.043 | -0.070 | 0.784 |
| Q33 | I regularly do exercise or walk for 30 to 40 minutes every day. | | **0.493** | -0.105 | -0.007 | 0.096 | 0.783 |
| Q28 | I eat fruit every day. | | **0.429** | -0.260 | 0.013 | 0.284 | 0.793 |
| Q30 | I eat breakfast every day | | **0.391** | 0.016 | 0.069 | -0.031 | 0.792 |
| Q40 | When visiting a doctor, I would tell him/her about my food allergic, if I had any. | | **0.313** | -0.101 | 0.119 | 0.113 | 0.800^a^ |
| Q67 | I manage my schedules in the way to be able to do exercise for half an hour every day. | | **0.245** | 0.152 | 0.004 | 0.214 | 0.791 |
| Q23 | I know how to find the responses of my questions. | | 0.207 | -0.025 | 0.117 | 0.189 | - |
| 1. **Interactive nutrition literacy** | | |  |  |  |  |  |
| Q48 | | I have enough will power to resist unhealthy foods (e.g., fast food, pizza, carbonated drinks, etc.) | 0.054 | **0.677** | -0.083 | 0.038 | 0.629 |
| Q50 | | If I go to restaurant or fast food with my friends, and all of them choose unhealthy foods (e.g., pizza, French fries, carbonated drinks, etc.), I’m able to choose healthy foods. | 0.083 | **0.622** | -0.082 | -0.029 | 0.627 |
| Q49 | | I can easily say “No” to any unhealthy eating suggestions from my friends. | 0.112 | **0.595** | -0.048 | 0.047 | 0.642 |
| Q44 | | If may family were overweight and eating a high fat diet, I would tell them -to change their eating habits. | -0.006 | **0.506** | 0.080 | -0.110 | 0.674 |
| Q43 | | If I encounter unhealthy behaviors at home, school or in other settings, I’m able to challenge them. | 0.165 | **0.489** | -0.008 | 0.124 | 0.639 |
| Q45 | | If my parents or family put unhealthy snack (e.g. chips, fruit roll, corn snack, etc.) for my school, I accept them. | 0.118 | **0.455** | -0.142 | 0.102 | 0.658 |
| Q36 | | I buy foods from street venders. | -0.088 | **0.378** | 0.092 | -0.071 | 0.797 ^a^ |
| Q24_1 | | When I go shopping with my mother or father, I buy healthy snacks such as nuts, raisins and dried chickpea, instead of chips, snacks, chocolate and sweets. | 0.032 | **0.349** | 0.258 | 0.124 | 0.671 |
| Q55 | | I trust media food advertising. | -0.114 | 0.307 | 0.129 | -0.189 | - |
| Q32 | | I add salt to my dish at the table. | -0.159 | 0.282 | 0.152 | -0.165 | - |
| Q53 | | Media food advertisings (e.g., TV, internet, radio, etc.) are not always correct. | -0.263 | 0.270 | 0.070 | 0.009 | - |
| Q59 | | If I was overweight, I would use high fat diet. | -0.146 | 0.256 | 0.178 | 0.193 | - |
| 1. **Food choice nutrition literacy** | | |  |  |  |  |  |
| Q24_6 | | When I go shopping with my mother or father, I buy foods that are certified as healthy. | 0.084 | 0.116 | **0.685** | -0.155 | .664 |
| Q24_4 | | When I go shopping with my mother or father, I buy foods that are not expired. | -0.176 | -0.012 | **0.609** | 0.052 | .689 |
| Q24_5 | | When I go shopping with my mother or father, I buy foods with standardized labeling. | 0.227 | 0.041 | **0.600** | -0.190 | .683 |
| Q24_3 | | When I go shopping with my mother or father, I buy foods with sustainable packaging. | -0.069 | 0.069 | **0.518** | 0.022 | .691 |
| Q24_2 | | When I go shopping with my mother or father, I buy foods that are stored appropriately or kept in refrigerator. | 0.033 | 0.019 | **0.459** | 0.103 | .689 |
| Q27 | | I eat food from all food groups every day. | 0.066 | -0.058 | **0.373** | 0.172 | .718 |
| 1. **Critical nutrition literacy** | | |  |  |  |  |  |
| Q60 | | I usually try new foods that I’ve never eaten. | -.084 | -.267 | .079 | **.519** | .401 |
| Q58 | | I can buy healthy food from the school cafeteria, depending on my pocket money. | -.002 | .209 | -.009 | **.469** | .428 |
| Q57 | | I usually try new vegetables that I’ve never eaten. | .001 | -.027 | .142 | **.409** | .320 |
| Q56 | | If the school cafeteria doesn’t offer any healthy foods, it will be difficult for me to choose healthy snack. | .094 | .013 | .102 | **-.385** | .489 |
| Q54 | | In spite of media food advertising, I can choose healthy foods depending on my needs. | -.021 | .193 | .138 | .264 | **-** |
| **Eigenvalue** | | | **6.78** | **2.51** | **2.07** | **1.55** | **-** |
| **Explained Variance (%)** | | | **17.03** | **5.11** | **3.76** | **2.24** | **-** |
| **Croanbach’s α** | | | **0.79** | **0.70** | **0.72** | **0.48** | **-** |
