# Supplementary material for: Developing and validating a scale to measure Food and Nutrition Literacy (FNLIT) in elementary school children in Iran
Source: PLoS One. 2017 Jun 27;12(6):e0179196. doi: 10.1371/journal.pone.0179196 (PMC5487019; doi:10.1371/journal.pone.0179196)
Supplement: S2 File — (PDF) [file pone.0179196.s006.pdf]

**پرسشنامه سنجش سواد غذا و تغذیه در کودکان ایرانی**
کد شناسایی دانش‌آموز: ..../...................../...................../........................
پایه تحصیلی: .... تاریخ بررسی: ..../............./...........

لطفاً سوالات را با دقت بخوانید و گزینه مناسب را انتخاب کنید.

ردیف	سوال
۱.	موقع خرید مواد غذایی، اطلاعات تغذیه ای درمورد مواد تشکیل دهنده آن ماده غذایی، چه قدر برای شما مهم است؟<br>الف) بسیار کم ب)کم ج)متوسط د)زیاد ه)خیلی زیاد
۲.	موقع خرید مواد غذایی، وجود علامت استاندارد بر روی بسته بندی ماده غذایی چه قدر برای شما مهم است؟<br>الف) بسیار کم ب)کم ج)متوسط د)زیاد ه)خیلی زیاد
۳.	موقع خرید مواد غذایی، تاریخ تولید و انقضاء بر روی بسته بندی ماده غذایی چه قدر برای شما مهم است؟<br>الف) بسیار کم ب)کم ج)متوسط د)زیاد ه)خیلی زیاد
۴.	من می توانم اطلاعات تغذیه ای (مانند مقدار انرژی، قند، چربی و ...) بر روی بسته بندی های مواد غذایی را به راحتی بفهمم.<br>الف- اصلا ب- خیلی کم ج- بعضی اوقات د- بیشتر اوقات ه- همیشه
۵.	من می توانم مطالب تغذیه ای که در روزنامه، مجلات و بروشورها چاپ می شود را به راحتی بفهمم.<br>الف- اصلا ب- خیلی کم ج- بعضی اوقات د- بیشتر اوقات ه- همیشه
۶.	من صحبت ها و توصیه های متخصصان تغذیه در مورد سلامت و نیازهای تغذیه ای متناسب با سن خودم را متوجه می شوم.<br>الف- اصلا ب- خیلی کم ج- بعضی اوقات د- بیشتر اوقات ه- همیشه
۷.	**آبپز** کردن از روشهای سالم پخت غذاست.<br>الف)کاملا صحیح ب) صحیح ج) نمی دانم د) غلط ه)کاملا غلط
۸.	من می توانم اطلاعات و توصیه های تغذیه ای مناسب برای کودکان که از رسانه ها مثل رادیو، تلویزیون و ... پخش می شود را بفهمم.<br>الف- اصلا ب- خیلی کم ج- بعضی اوقات د- بیشتر اوقات ه- همیشه
۹.	روزانه ۳۰ تا ۴۰ دقیقه ورزش منظم موجب جلوگیری از چاقی می شود.<br>الف)کاملا صحیح ب) صحیح ج) نمی دانم د) غلط ه)کاملا غلط
۱۰.	من می دانم که سبزیهای مختلف چگونه کاشته می شوند و رشد می کنند.<br>الف) خیلی زیاد ب) زیاد ج) کم د) خیلی کم ه) نمی دانم
۱۱.	مصرف تنقلات شور مثل چیپس و پفک برای سلامتی مضر است.<br>الف)کاملا صحیح ب) صحیح ج) نمی دانم د) غلط ه)کاملا غلط
۱۲.	مصرف زیاد قند، شکر، شیرینی و شکلات برای سلامتی مضر است.<br>الف)کاملا صحیح ب) صحیح ج) نمی دانم د) غلط ه)کاملا غلط
۱۳.	سوسیس و کالباس، چربی بالایی دارند و ممکن است باعث چاقی شوند.<br>الف)کاملا صحیح ب) صحیح ج) نمی دانم د) غلط ه)کاملا غلط
۱۴.	مصرف سوسیس و کالباس احتمال خطر سرطان را زیاد می کند.<br>الف)کاملا صحیح ب) صحیح ج) نمی دانم د) غلط ه)کاملا غلط

۱۵.	خواندن تاریخ تولید و انقضا روی بسته بندی مواد غذایی برای سلامتی مهم است.<br>الف)کاملا صحیح ب) صحیح ج) نمی دانم د) غلط ه)کاملا غلط
۱۶.	من هر روز سبزیهای مختلف مانند کاهو، کلم، گوجه، هویچ و ... می خورم.<br>الف- اصلا ب- خیلی کم ج- بعضی اوقات د- بیشتر اوقات ه- همیشه
۱۷.	من می توانم در مورد مطالب تغذیه ای که از منابع مختلف به دست آورده ام با دیگران (دوستانم، افراد خانواده، فامیل و...) صحبت کنم.<br>الف- اصلا ب- خیلی کم ج- بعضی اوقات د- بیشتر اوقات ه- همیشه
۱۸.	من در مورد تغذیه سالم با دوستان و افراد خانواده ام گفتگو می کنم.<br>الف- اصلا ب- خیلی کم ج- بعضی اوقات د- بیشتر اوقات ه- همیشه
۱۹.	اگر برایم سوالی در مورد غذا و تغذیه پیش بیاید از پدر مادر، معلم و یا دیگران پرس و جو می کنم.<br>الف- اصلا ب- خیلی کم ج- بعضی اوقات د- بیشتر اوقات ه- همیشه
۲۰.	من برای خوراکی مدرسه، **خودم** لقمه درست می کنم.<br>الف- اصلا ب- خیلی کم ج- بعضی اوقات د- بیشتر اوقات ه- همیشه
۲۱.	من میان وعده یا لقمه های غذایی سالم به مدرسه می برم.<br>الف- اصلا ب- خیلی کم ج- بعضی اوقات د- بیشتر اوقات ه- همیشه
۲۲.	من بطور منظم، روزانه ۳۰ تا ۴۰ دقیقه، ورزش یا پیاده روی می کنم.<br>الف- اصلا ب- خیلی کم ج- بعضی اوقات د- بیشتر اوقات ه- همیشه
۲۳.	من میوه و سبزیها را، **خودم** می شویم و آماده می کنم.<br>الف- اصلا ب- خیلی کم ج- بعضی اوقات د- بیشتر اوقات ه- همیشه
۲۴.	من هر روز میوه می خورم.<br>الف- اصلا ب- خیلی کم ج- بعضی اوقات د- بیشتر اوقات ه- همیشه
۲۵.	من هر روز صبحانه می خورم.<br>الف- اصلا ب- خیلی کم ج- بعضی اوقات د- بیشتر اوقات ه- همیشه
۲۶.	من می توانم در برابر تمایلات و هوس هایم به غذاهای ناسالم مانند فست فود، پیتزا، نوشابه و ... مقاومت کنم و نخورم.<br>الف- اصلا ب- خیلی کم ج- بعضی اوقات د- بیشتر اوقات ه- همیشه
۲۷.	اگر با دوستانم به رستوران یا فست فود فروشی بروم و همه آنها غذاهای ناسالم سفارش دهند، من غذاهای سالم تر را انتخاب می کنم.<br>الف- اصلا ب- خیلی کم ج- بعضی اوقات د- بیشتر اوقات ه- همیشه
۲۸.	من به راحتی می توانم در برابر پیشنهادهای دوستانم در مورد مصرف خوراکی های ناسالم **"نه"** بگویم.<br>الف- اصلا ب- خیلی کم ج- بعضی اوقات د- بیشتر اوقات ه- همیشه
۲۹.	اگر رفتارهای تغذیه ای ناسالم را در خانه، مدرسه یا محیط های دیگر ببینم می توانم با آنها مقابله کنم.<br>الف- اصلا ب- خیلی کم ج- بعضی اوقات د- بیشتر اوقات ه- همیشه
۳۰.	اگر مادر، پدر یا سایر افراد خانواده، خوراکی های ناسالم مانند چیپس، لواشک و... برای میان وعده مدرسه ام بگذارند، قبول می کنم.<br>الف- اصلا ب- خیلی کم ج- بعضی اوقات د- بیشتر اوقات ه- همیشه
۳۱.	اگر افراد خانواده ام اضافه وزن داشته باشند و از مواد غذایی پرچربی استفاده کنند به آنها می گویم که این غذاها را نخورند.<br>الف- اصلا ب- خیلی کم ج- بعضی اوقات د- بیشتر اوقات ه- همیشه
۳۲.	اگر با مادر یا پدرم برای خرید به فروشگاه بروم و یا از بوفه مدرسه خرید کنم، به جای چیپس، پفک، شکلات و شیرینی، تنقلات

	سالم مانند مغزدانه ها، نخودچی کشمش و خشکبار می خرم.<br>الف- هیچوقت ب- خیلی کم ج- بعضی اوقات د- بیشتر اوقات ه- همیشه
۳۳.	اگر با مادر یا پدرم برای خرید به فروشگاه بروم و یا از بوفه مدرسه خرید کنم، خوراکیهایی را می خرم که پروانه ساخت وزارت بهداشت داشته باشد.<br>الف- هیچوقت ب- خیلی کم ج- بعضی اوقات د- بیشتر اوقات ه- همیشه
۳۴.	اگر با مادر یا پدرم برای خرید به فروشگاه بروم و یا از بوفه مدرسه خرید کنم، خوراکیهایی را می خرم که مهر استاندارد داشته باشد.<br>الف- هیچوقت ب- خیلی کم ج- بعضی اوقات د- بیشتر اوقات ه- همیشه
۳۵.	اگر با مادر یا پدرم برای خرید به فروشگاه بروم و یا از بوفه مدرسه خرید کنم، خوراکیهایی را می خرم که تاریخ مصرفشان نگذشته باشد.<br>الف- هیچوقت ب- خیلی کم ج- بعضی اوقات د- بیشتر اوقات ه- همیشه
۳۶.	اگر با مادر یا پدرم برای خرید به فروشگاه بروم و یا از بوفه مدرسه خرید کنم، خوراکیهایی را می خرم که دارای بسته بندی باشد.<br>الف- هیچوقت ب- خیلی کم ج- بعضی اوقات د- بیشتر اوقات ه- همیشه
۳۷.	اگر با مادر یا پدرم برای خرید به فروشگاه بروم و یا از بوفه مدرسه خرید کنم، خوراکیهایی را می خرم که در جای مناسب، مثل یخچال یا قفسه نگهداری شده باشند.<br>الف- هیچوقت ب- خیلی کم ج- بعضی اوقات د- بیشتر اوقات ه- همیشه
۳۸.	من در طول روز از همه گروههای غذایی استفاده می کنم.<br>الف- اصلا ب- خیلی کم ج- بعضی اوقات د- بیشتر اوقات ه- همیشه
۳۹.	من معمولا غذاهای جدیدی که قبلا نخورده ام را امتحان می کنم.<br>الف) اصلا ب) خیلی کم ج) بعضی اوقات د) بیشتر اوقات ه) همیشه
۴۰.	من معمولا سبزیهای جدیدی که قبلا نخورده ام را امتحان می کنم.<br>الف) اصلا ب) خیلی کم ج) بعضی اوقات د) بیشتر اوقات ه) همیشه
۴۱.	می توانم به تناسب پول توجیبی ام، غذاهایی را از بوفه/مغازه بخرم که مفید باشند.<br>الف) اصلا ب) خیلی کم ج) بعضی اوقات د) بیشتر اوقات ه) همیشه
۴۲.	وقتی بوفه مدرسه/مغازه مواد غذایی سالم ندارد، انتخاب یک خوراکی مفید از بوفه/مغازه برایم مشکل می شود.<br>الف) اصلا ب) خیلی کم ج) بعضی اوقات د) بیشتر اوقات ه) همیشه

۴۳ .آیا تا به حال جدول اطلاعات تغذیه ای بر روی بسته بندی مواد غذایی را دیده اید؟

الف) بلی ب) خیر

۴۴. دو جدول زیر نشان دهنده اطلاعات تغذیه ای بر روی دوپاکت شیر می باشند، با توجه به اطلاعات درج شده، کدامیک از این دو محصول را انتخاب می کنید؟در صورت انتخاب هر کدام از این محصول،دلیل خود را بنویسید.

جدول حقایق تغذیه ای		
کالری در هر ۱۰۰ گرم ماده غذایی ۵۲/۹۲ کیلوکالری		
کالری حاصل از چربی در هر ۱۰۰ گرم ماده غذایی ۲۸/۸ کیلوکالری		
ارزش روزانه (DV ٪) در هر ۱۰۰ گرم	اطلاعات تغذیه ای	
۴/۸۸	چربی کل برحسب گرم	۳/۲
۱۰	اسیدهای چرب اشباع بر حسب گرم	۲
۰	اسیدهای چرب ترانس برحسب گرم	۰
۴/۵	پروتئین بر حسب گرم	۳/۱۵
۱	کربوهیدرات کل بر حسب گرم	۲/۸۸
۰/۱	قند بر حسب گرم	۰/۳۶
۱/۷۵	سدیم بر حسب میلی گرم	۴۲
*ارزش روزانه بر اساس رژیم غذایی ۲۰۰۰ کیلوکالری		
چربی کل کمتر از ۶۵ گرم<br>اسیدهای چرب اشباع کمتر از ۲۰ گرم<br>سدیم کمتر از ۲۴۰۰ میلی گرم<br>کربوهیدرات ۳۰۰ گرم		

شکل الف)

جدول حقایق تغذیه ای<br>تعداد سهم در هر بسته: ۴<br>اندازه سهم: ۲۳۰ گرم		
مقدار در هر سهم<br>انرژی: ۸۷/۴ Kcal		
%مقدار مرجع ماده مغذی (NRV)	اطلاعات تغذیه ای	
**	چربی کل (گرم)	۲/۷۶
**	کربوهیدرات (گرم)	۸/۵۱
**	قند (گرم)	۰/۲۷
٪ ۱۴/۲۶	پروتئین (گرم)	۷/۱۳
نباید زیاد مصرف شود:		
%۹/۲	اسیدهای چرب اشباع (گرم)	۱/۸۴
**	اسیدهای چرب ترانس (گرم)	۰
%۱۲/۱۲	نمک سدیم (میلی گرم)	۲۴۲,۶۵
*NRV تعیین نگردیده است.		

شکل ب)

الف) ب) ج) نمی دانم

– چرا؟ (توضیح دهید.)..

۴۵. با توجه به اطلاعات رنگی شکل مقابل که مربوط به تافی (یک نوع شکلات) است، به سوالات زیر پاسخ دهید.

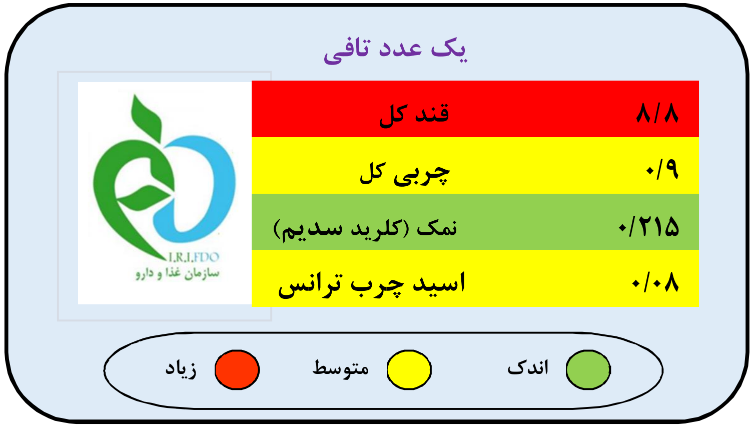

۱- به نظر شما مقدار قند در این محصول چگونه است؟

الف) کم ب)متوسط ج) زیاد د)نمی دانم

۲- به نظر شما مقدار چربی کل و اسید چرب ترانس در این محصول چگونه است؟

الف) کم ب)متوسط ج) زیاد د)نمی دانم

۳- به نظر شما مقدار نمک در این محصول چگونه است؟

الف) کم ب)متوسط ج) زیاد د)نمی دانم

۴۶. با توجه به اطلاعات رنگی شکل مقابل که مربوط به شیر پاستوریزه است، به سوالات زیر پاسخ دهید.

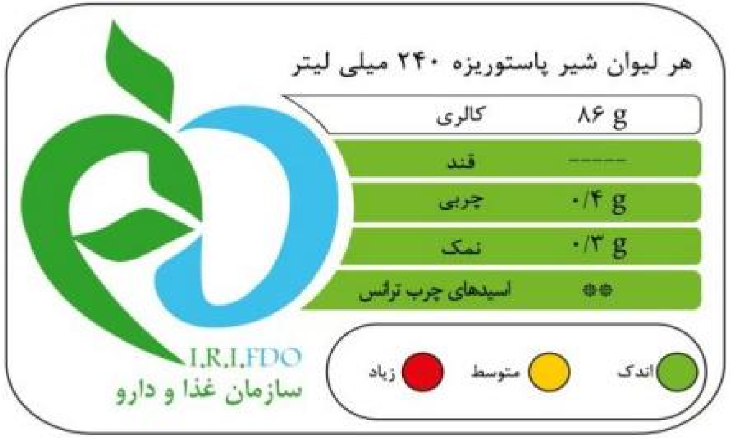

۱- به نظر شما مقدار چربی در این محصول چگونه است؟

الف) کم ب)متوسط ج) زیاد د)نمی دانم

۲- به نظر شما مقدار نمک در این محصول چگونه است؟

الف) کم ب)متوسط ج) زیاد د)نمی دانم

© Doustmohammadian. et al., 2016
